# Erratum: P53-regulated autophagy and its impact on drug resistance and cell fate

**DOI:** 10.20517/cdr.2021.97

**Published:** 2021-09-23

**Authors:** Daeun Shim, Lei Duan, Carl G. Maki

**Affiliations:** Department of Cell and Molecular Medicine, Rush University Medical Center, Chicago, IL 60612, USA.

The original article was published on 19 Mar 2021, and the original article link is https://cdrjournal.com/article/view/3789

The corresponding author declared that The Department of Defense Grant support acknowledgment was incorrectly entered. The Department of Defense Grant award that helped support this work was W81XWH-16-1-0025. Therefore, he needed to change financial support and sponsorship in his publication in our journal.

According to the evidence provided by the corresponding author, this work was supported by the National Cancer Institute Grant (R01CA200232-05) and the Department of Defense Grant (W81XWH-16-1-0025) both to Maki CG, and all of the other authors of this paper agreed with the change mentioned above. Therefore, we publish this erratum to announce this change.

Cite this article: Shim D, Duan L, Maki CG. P53-regulated autophagy and its impact on drug resistance and cell fate. *Cancer Drug Resist* 2021;4:85-95. http://dx.doi.org/10.20517/cdr.2020.85

